# Retraction Note: mTORC2 regulates hedgehog pathway activity by promoting stability to Gli2 protein and its nuclear translocation

**DOI:** 10.1038/s41419-024-06723-5

**Published:** 2024-05-21

**Authors:** Samarpan Maiti, Susmita Mondal, Eswara M. Satyavarapu, Chitra Mandal

**Affiliations:** Cancer Biology and Inflammatory Disorder Division, Council of Scientific and Industrial Research (CSIR)-Indian Institute of Chemical Biology, 4, Raja S.C. Mullick Road, Jadavpur, Kolkata, 700032 India

Retraction to: *Cell Death and Disease* 10.1038/cddis.2017.296, published online 13 July 2017

The Editors-in-Chief have retracted this article. After publication, concerns were raised regarding some of the data presented in the figures, specifically:

- In Fig. 3g, two pairs of 0h images (U87MG shRictor and Hedgehog inhibitor; LN229 Rictor overexpressed and Rictor overexpressed + Hedgehog inhibitor) appear to overlap.
- Fig. 2f Ptch1 lane 1 appears highly similar to Fig. 4b LN229 Gli2-FL lane 3.
- Fig. 4e Cyclin D1 blot appears highly similar to Fig. 5a IP: mTOR IB: Rictor.
- Fig. 5e Oct4 blot appears highly similar to Supplementary Fig. S2c Sox2.

The authors have stated that these images were selected by mistake during final figure preparation. Due to the number of errors in the figures, the Editors-in-Chief no longer have confidence in the presented data.

Chitra Mandal does not agree to this retraction. None of the other authors have responded to any correspondence from the publisher about this retraction.

